# Uniaxial Compression Mechanical Properties of Foam Nickel/Iron-Epoxy Interpenetrating Phase Composites

**DOI:** 10.3390/ma14133523

**Published:** 2021-06-24

**Authors:** Xiaoxing Wang, Yu Zhou, Jingli Li, Huijian Li

**Affiliations:** School of Civil Engineering and Mechanics, Yanshan University, Qinhuangdao 066004, China; FREUD1224@163.com (X.W.); zyjlwelcome@163.com (Y.Z.); ljlwelcome@163.com (J.L.)

**Keywords:** interpenetrating phase composites, uniaxial compression experiments, mechanical properties, energy absorbing, stress relaxation, creep deformation

## Abstract

The damage process and failure mechanisms were analyzed by a series of quasi-static compressive experiments of seven materials including pure epoxy (EP), three different PPI (pores per linear inch) foam nickel-iron, and three different PPI foam nickel/iron-epoxy interpenetrating phase composites (IPC). Plotting the stress–strain curves of different materials, their change rules are discussed, then the effective elastic modulus and yield limit of the materials are provided, and the energy absorption properties of different materials are analyzed by the stress–strain curves. It was found that the effective elastic modulus and specific stiffness of the three IPC materials were higher than pure foam nickel-iron. The brittleness of epoxy can be obviously changed by selecting a suitable PPI foam nickel-iron composited with it. The unit volume energy absorption rate of foam nickel/iron-epoxy was significantly higher than pure epoxy and pure foam nickel-iron. It was also found that the energy absorption rate decreased with the increase in PPI. The stress relaxation rate decreased first and then increased with the increase in PPI. The creep behavior of the three composites was obvious in the creep elastic stage, and the creep rate increased with the increase in PPI. The creep rate decreased with the increase in PPI in the creep transition stage.

## 1. Introduction

The porous metal foam material has the advantages of sound absorption, shock absorption, light weight, heat resistance, and high strength, etc., and has been widely used in machinery, chemistry, construction, aerospace, biomedicine, and other fields [1]. This microstructure is composed of a framework and internal pores make it have both the properties of metal materials and foam materials. With the continuous progress in technology, the demand for such materials in engineering is more urgent. In addition to updating and optimizing the existing materials, it is also necessary to study more novel materials. The new materials of interpenetrating phase composites (IPC) have attracted the attention of researchers. The traditional composite material is through the ingenious combination of two or more different components to achieve material properties that each component cannot play in a single state [2,3]. From the perspective of morphological structure, the composite material is a composite of a continuous phase and one or more dispersed phases, or a composite of two or more continuous phases and one or more dispersed phases in each continuous phase; usually the continuous phase is called the matrix and the dispersed phase is the filler, which plays the role of strengthening, toughening, stiffening, or other functional properties. Interpenetrating phase composites (IPC) are a new kind of material with a special meso-structure. Each component phase forms its own three-dimensional network structure on the meso-scale and interweaves with each other. Even if any component phase in the material is removed, the remaining component phase can still form an open cell pore structure that can withstand an external load. IPC is different from traditional composite materials such as foam Ni/Fe-EP IPC materials that combine the characteristics of metal and organic matter can be designed as a high strength, low stiffness, and high viscosity material. Furthermore, it keeps the foam metal electromagnetic shielding properties and low thermal conductivity of an epoxy. IPC can show its own characteristics on a macro scale. According to the actual engineering needs, it can design a composite material that not only meets certain mechanical properties, but also has one or more functions. This new material can be used for anti-collision materials that are often applied in aerospace, transportation, and other fields. It can also be a new type of damping material as well as a new type of electromagnetic shielding and vibration isolation building material.

A composite foam material filled with epoxy resin in the foam nickel-iron is a new type of IPC material. It has been found that IPC obtained by interpenetrating foam metal and epoxy resin can effectively improve the mechanical properties of the material. Yu Wei et al. [4] studied the deformation process and failure appearance of pure aluminum foam, pure epoxy resin, and three kinds of hollow glass microspheres of an aluminum foam-epoxy resin interpenetrating phase composites with different volume fraction, and found that the effective elastic modulus, yield strength, specific strength, and specific stiffness of three kinds of IPC were significantly improved compared with the pure aluminum foam. The aluminum foam-epoxy resin had the highest energy absorption rate per unit volume. Bilal Mansoor et al. [5] prepared an aluminum foam with rolled opening 6101 and studied the effects of different relative densities and strain rates on its compression behavior. The results showed that the compression platform strength of the foam increased with the increase in relative density, and different strain rates had little effect on its compression behavior. Nihad Dukhan et al. [6] prepared aluminum foam-polypropylene interpenetrating phase composites by the injection molding method, and studied the bending properties of the composites. The results show that the flexural modulus and flexural strength of the interpenetrating phase composites were greatly improved compared with that of the pure aluminum foam, and the flexural stiffness of the interpenetrating phase composites increased with the decrease in the aperture of aluminum foam. Long Jing et al. [7] studied the aging resistance of aluminum powder-epoxy resin composite materials and found that the addition of aluminum powder could effectively block the entry of corrosive media. With the increase in the amount of flake aluminum powder, the better the corrosion resistance of the material. Wouterson et al. [8] analyzed the influence of the filling amount and length of carbon fiber on the mechanical properties of carbon fiber reinforced composite foam materials, and the experiment found that the filling of carbon fiber could significantly enhance the mechanical properties of the composite materials such as tensile strength and elastic modulus, and the toughening effect was good. Idris et al. [9] prepared closed cell aluminum foam panels by the direct foaming method and studied their mechanical behavior and energy absorption capacity under uniaxial compression. The results showed that the densification strain of the foam decreased with the increase in the foam density, while the energy absorption capacity increased with the increase in the foam density. Jhaver and Tippur [10] prepared two kinds of HGB and epoxy resin composite foams with different volume fraction and aluminum composite IPC material, and studied its effective elastic modulus and yield stress. They found that its performance was better than pure composite foams. Li Xiang et al. [11] prepared an epoxy resin-hollow glass microsphere composite foam material and studied its compression failure form, density, microstructure, and curing process. Pathak et al. [12] modified the epoxy resin with graphene oxide and filled the carbon fiber into the modified resin matrix. The addition of graphene oxide significantly enhanced the interfacial force between the carbon fiber and the resin matrix and improved the overall mechanical properties of the material. Yu Yinghua et al. [13] studied the compressive properties of foamed aluminum-epoxy resin composites through finite element simulation analysis, which proved the feasibility of the simulation method and provided a new way for the study of the performance of aluminum foam-cavity filled composites. Tilbrook et al. [14] studied the mechanical properties and fatigue properties of aluminum-epoxy composites and found that IPC materials had better performance. Jiang Shengli et al. [15] prepared a foam nickel-epoxy-silicon carbide bi-continuous composite material and studied its damage behavior under the erosion condition of sodium chloride slurry. Mohammad et al. [16] prepared an aluminum foam sandwich structure and used LS-DYNA software to simulate the energy absorption capacity of the aluminum foam sandwich structure with different relative densities under the condition of the drop hammer experiment. The results show that the energy absorption capacity is related to the relative density of aluminum foam. Xie Beiping et al. [17] studied the damping properties of carbon nanotube modified foam nickel-iron-epoxy resin composites. It can be seen that there have been few research studies on foam nickel/iron-epoxy IPC. Compared with other porous metal materials, the foam nickel/iron alloy has the advantages of rich resources, low price, and high strength. It is necessary to conduct an in-depth study of the basic mechanical properties, dynamic characteristics, viscoelasticity, and other properties of foam nickel/iron-epoxy IPC. Through a series of experiments, the author of this paper conducted research and analysis on the effective elastic modulus, yield limit, energy absorption performance, stress relaxation, and creep of foam nickel/iron-epoxy IPC.

## 2. Materials and Methods

### 2.1. Materials

**Figure 1:**
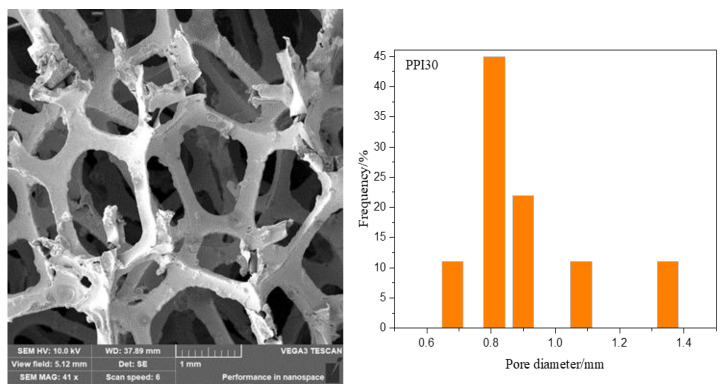
SEM image and pore diameter distribution of PPI30.

**Figure 2:**
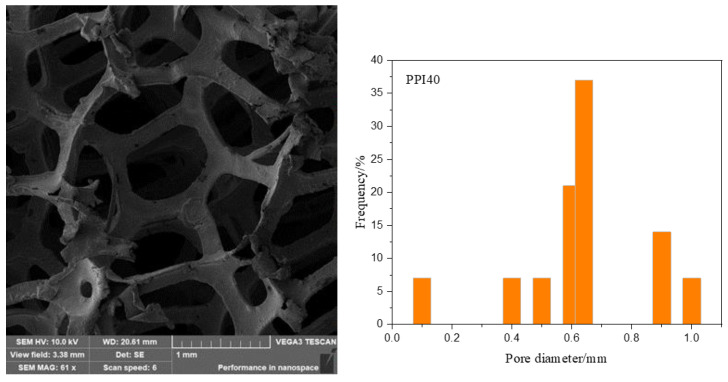
SEM image and pore diameter distribution of PPI40.

The foam nickel-iron used in the experiment was an open-cell foam nickel-iron. The pore sizes of the three foam nickel-iron were PPI20, PPI30, and PPI40 (the penetration of pores and the integrity of pores and ribs are the key factors to form good interpenetrating IPC materials. Restricted by the current production process, the number of fractured ribs increases when PPI is less than 20, and the penetration of pores is limited when PPI is greater than 40, so we chose PPI20, PPI30m and PPI40 as the experimental objects), and their apparent densities are 0.18 g/cm^3^, 0.21 g/cm^3^, and 0.26 g/cm^3^, respectively. SEM (TESCON, Brno, Czech Republic) images and the figures of the pore diameter distribution for PPI30 and PPI40 are shown in Figure 1 and Figure 2. The epoxy resin was bisphenol A E44, and the curing agent was a polyamide active curing agent 651. The plasticizer was dibutyl phthalate, and the defoamer was dimethyl silicone oil.

### 2.2. Preparation of Composite Materials

**Figure 3:**
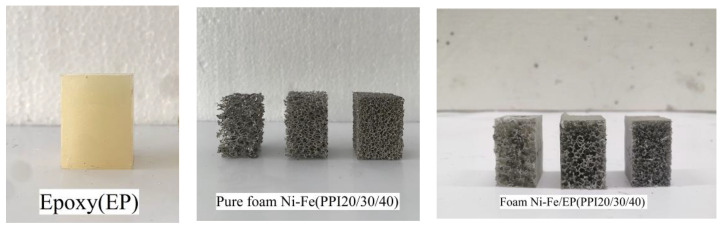
Compression specimen of the pure epoxy, foam Ni-Fe, and foam Ni-Fe/EP interpenetrating phase composites.

First, the foam nickel-iron was processed into a rectangular specimen of 20 mm × 20 mm × 30 mm, and the processed foam nickel-iron specimen was cleaned with industrial alcohol and dried to remove all impurities in the holes of the foam nickel-iron specimen during the preparation process. Then, two plastic beakers were respectively used to fill a certain amount of epoxy resin and curing agent with a 1:1 volume ratio, and put into a constant temperature water bath to heat for a period of time to increase their fluidity. In order to enhance the wetting property of the epoxy resin, a 2% volume defoamer was added to the epoxy resin. The defoaming agent was poured into the epoxy resin and stirred for about 15 min to ensure that the bubbles in the epoxy resin were fully discharged. Then, the heated curing agent was poured into the epoxy resin and stirred quickly. The mixed liquid was put into the vacuum pump to remove bubbles, again to ensure that the bubbles in the mixed liquid were completely discharged. Then, the mixture was poured into the mold to be fully soaked with the foam nickel-iron, while a single piece of pure epoxy resin of the same size was poured. After curing at 25 °C for a week, the mold was removed, the specimen was taken out, the excess epoxy resin on the surface of the specimen was removed, and the two ends of the specimen were polished and leveled with sandpaper to meet the experimental requirements. In addition, for stress relaxation and creep specimens, plasticizer with a volume ratio of 2% was added in the preparation process of epoxy resin. The PPI used in the experiment in this paper were 20/30/40, respectively. According to the purpose of the experiment, six composite material specimens under each PPI were made, three pure foam nickel-iron specimens under each PPI were made, and three pure epoxy resin specimens were made. The specimens were grouped and numbered for reserve. Figure 3 shows the seven specimens used in the experiment, which were pure epoxy resin, foam nickel-iron with PPI 20/30/40, and foam nickel-iron/epoxy resin composite with PPI 20/30/40, respectively.

### 2.3. Compressive Test

**Figure 4:**
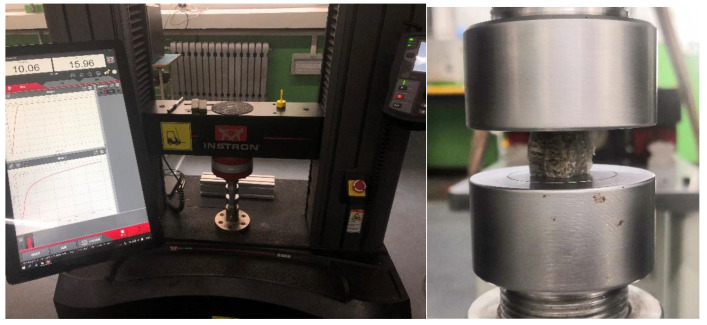
Experimental equipment and close-up of the specimen.

The prepared specimens were subjected to compression experiments at 25 °C with the experimental instrument of an INSTRON testing machine (Model No. 5982, INSTRON, Norwood, MA, USA), and the loading rate was 1 mm/min. All experimental data were automatically collected by computer. The calculation of mechanical properties followed these standards. For the compressive test of pure epoxy (EP) and foam nickel/iron-epoxy interpenetrating phase composites (IPC), we referred to the GB/T 2569 (China). For the compressive test of foam nickel-iron, we referred to ISO 13314. For the compressive test of pure Ni/Fe alloy, we referred to ASTM E9-09. For the creep test, we referred to ISO 7850. The experimental equipment and a close-up of the specimen are shown in Figure 4.

## 3. Results

### 3.1. Analysis of Deformation and Failure Morphology

**Figure 5:**
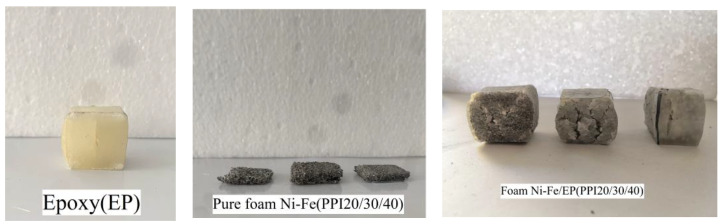
Damage morphologies of the pure epoxy, foam Ni-Fe, and foam Ni-Fe/EP interpenetrating phase composites.

As can be seen from Figure 5, pure foam Ni-Fe was finally compressed into a “biscuit” shape after undergoing the linear elastic compression stage, plastic yield stage, and pore wall compaction stage, which was in line with the general deformation morphology law presented by foam material compression [18]. Both epoxy resin and foam Ni-Fe/EP resin composite specimens first appeared as a linear elastic compression stage, plastic yield stage, and finally compression failure in the shape of a waist drum during the compression process. Oblique cracks existed on the four sides of the specimen, and vertical cracks appeared in the middle area of some specimens. The appearance of the compression failure form of this straight crack was related to the friction between the indenter of the testing machine and the upper and lower surfaces of the test piece. For the failure of straight cracks, first, after elastic yielding, cracks appeared at the defect positions in the middle of the four sides of the specimen. As the compressive stress increased, the friction between the squeeze head of the experimental equipment and the upper and lower surfaces of specimen also increased. The combination of these two forces makes the material yield condition follow the fourth strength theory, which macroscopically showed that the crack growth was caused by the tensile stress perpendicular to the crack. The crack damage belonged to the I-shaped crack failure. The crack propagated to the middle of the specimen, and the area of the core compression zone became smaller and smaller. Finally, the crack penetrated and the specimen was destroyed. The failure of oblique cracks occurred after elastic yielding, and cracks appeared in the middle of the four sides of the specimen where some minor defects appeared. As the compressive stress increased, the friction caused by the squeeze head of the experimental equipment was small and the shear stress of the material reached the yield stress. It followed the third strength theory, which macroscopically showed that the crack growth was caused by the shear stress parallel to the crack. The crack damage belonged to type II crack damage. The crack gradually expanded to the middle of the specimen, and finally, the crack penetrated and the specimen was destroyed. The difference was that epoxy cracks first appeared at the micro-defects in the middle of the side of the specimen, while the foam Ni-Fe/EP IPC material first appeared at the defects at the interface between the epoxy resin and the foam nickel-iron. Due to the different Young’s modulus of the two materials, in order to maintain the same strain at the interface, the two materials had shear stress at the interface. With the continuous expansion of the shear stress, the crack developed. At the same time, due to the different deformation modulus, an interlocking effect will form inside the interpenetrating material, which is beneficial to improve the compressive or tensile bearing capacity of the composite. Epoxy resins with high brittleness after curing exhibit the characteristics of brittle materials when broken. As a typical IPC material, foam Ni-Fe/EP IPC material has the properties of both materials. After the composite, the original properties of each component can be brought into play. Therefore, in the process of the experiment, it showed the characteristics of the plastic material and brittle material when damaged. Therefore, this kind of IPC composite can improve the mechanical properties of the materials by taking advantage of the advantages of each other.

### 3.2. Mechanics Property Analysis

**Figure 6:**
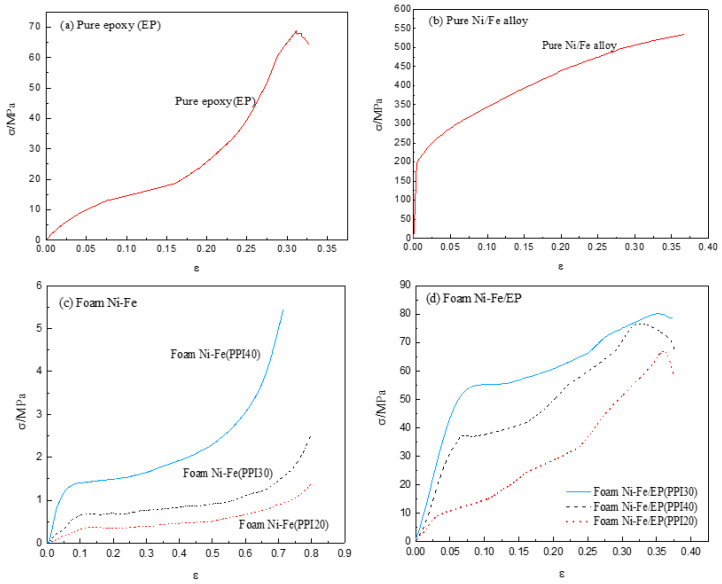
Stress–strain curves of pure epoxy (**a**), pure Ni-Fe (**b**), pure foam Ni-Fe (**c**), and foam Ni-Fe/EP interpenetrating phase composites (**d**).

Figure 6c shows the stress–strain curve of the quasi-static compression process of foam Ni-Fe with three different PPI. It can be clearly seen from the curve shown in the figure that, except for the very small nonlinear stage at the beginning of the experiment, the foam Ni-Fe exhibited a three-stage rule in the compression process: the linear elastic stage, the plastic yield platform stage, and the compact stage. The elastic stage of the three different PPI all ended at about 10% strain, while the platform stage was slightly different. With the increase in PPI, the strain at the end of the platform stage decreased. The PPI40 foam Ni-Fe platform stage ended at about 30% strain, PPI30 at about 40% strain, and PPI20 at about 45% strain. It can also be seen from the stress–strain curve that with the increase in PPI, the slope in the linear elastic stage also increased, indicating that its elastic modulus increased. According to Equation (1), the elastic modulus of the foam Ni-Fe material is proportional to the mechanical properties of its base material [18]:

(1) $$\frac{E^{*}}{E_{s}}=C_{1}{\left(\frac{\rho^{*}}{\rho_{s}}\right)}^{2}$$

In Equation (1), E_s_ and ρ_s_ represent the elastic modulus and density of the matrix material, respectively; E* and ρ* represent the elastic modulus and density of the foam material, respectively; and C_1_ is the proportionality constant. According to the calculation of the experimental results, C_1_ of the foam nickel-iron material in the formula can be 0.14, as shown in Table 1. With the increase in PPI, the density ρ* of the foam material increased, and the elastic modulus E^*^ of the foam material also increased.

The force–displacement curve was obtained by the experimental equipment, and the displacement was divided by the initial specimen height to obtain the strain, as shown in Equation (2). The stress was obtained by dividing the force by the initial specimen section area, as shown in Equation (3).

(2) $$\varepsilon =\frac{\Delta l}{l_{0}}$$

(3) $$\sigma =\frac{F}{A_{0}}$$

In Equation (2), *ε* is strain and ∆*l* and *l*_0_ represent the compressive displacement of the specimen and the initial height of the specimen, respectively. In Equation (3), *σ* is stress, and *F* and *A*_0_ represent force and initial section area of specimen, respectively.

**Figure 7:**
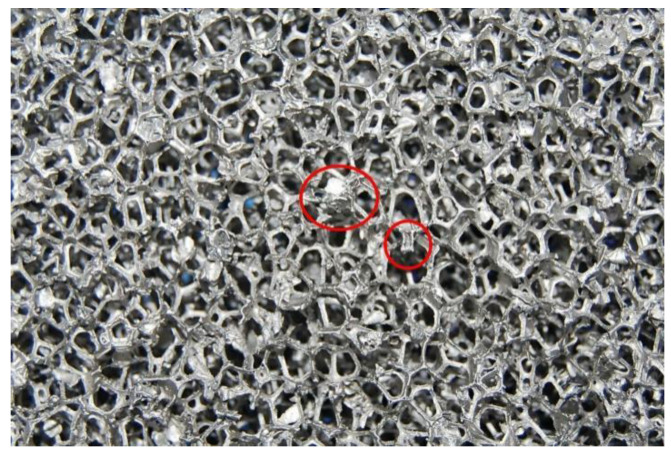
Local view of foam Ni-Fe.

As shown in Figure 6d, the stress–strain curve of IPC filled with epoxy resin in foam nickel-iron showed different changing rules with different PPI. IPC of PPI20 in the quasi-static compression test showed three stages similar to the epoxy resin and its platform stage was very short, an approximation from the linear elastic stage directly into the plastic growth stage. We believe that the reason for this phenomenon is, first, the porosity of the PPI20 foam nickel-iron was larger, the number of prisms that provided support was less, and most of its cross-section area was the epoxy resin part. The second is that some invalid prisms formed during the processing. As shown in Figure 7, these prisms had no significant increase in the bearing capacity of the compression during the compression process. Third, due to the limitation of the number of prisms, the effect of improving the bearing capacity caused by the interlocking effect of the foam nickel-iron and epoxy resin was very limited. Therefore, the performance of IPC material in quasi-static compression was similar to that of pure epoxy resin. With the increase in strain, the strain of the composite material increased and cracks expanded rapidly, then the bearing capacity significantly reduced and brittle failure occurred. IPC of PPI30 and PPI40 showed four stages, namely the elastic stage, plastic platform stage, plastic growth stage, and failure stage. The reason is that with the increase in PPI, the porosity decreased, the area of Fe-Ni in the cross section increased, the number of prisms increased, the filling amount of epoxy resin decreased, and the high strength and other metallic properties of Fe-Ni were gradually shown, thus improving the toughness of the composites and prolonging the yield platform stage. By comparing the two figures, it was obvious that the mechanical properties of three different PPI composites were greatly improved compared with the pure foam Fe-Ni. In the test, the elastic modulus and yield limit of PPI30 IPC were higher than that of PPI40.We think that when PPI is 40, due to the lack of wetting, micro-cracks and bubbles are more likely to form on the interface between foam Ni-Fe and epoxy resin. Compared with the strengthening of the bearing capacity caused by the interlocking effect, the damage caused by micro-cracks on the interface had a greater impact on the mechanical properties of IPC materials. Therefore, attention should be paid to the wetting of the interface in the production process of this material.

Table 2 shows the mechanical property data of seven materials. As can be seen from the table, the elastic modulus of IPC material with PPI30 was the largest, while that of foam Ni-Fe was the smallest. The elastic modulus of IPC material with PPI20 was similar to that of epoxy resin. The mechanical properties of the three kinds of IPC materials first increased and then decreased with the increase in PPI. Among them, the PPI30 and 40 IPC materials showed an obvious plastic yield platform during the compression process, which was not found in the compression stress–strain curve of pure epoxy resin. This shows that choosing the right PPI and combining with epoxy resin can significantly improve the toughness of the epoxy resin. In addition, the specific stiffness of the composites with PPI30 and 40 was higher than that of the foam materials and pure epoxy resin, indicating that the composites had both the properties of the two materials. It can be seen that a new type of material can be obtained by the composite of epoxy resin and foam Ni-Fe to meet more practical needs. Besides, by changing the PPI size of foam Ni-Fe, the mechanical properties of the composite materials can be different, so as to expand the application range of composites.

### 3.3. Energy Absorption Performance Analysis

The energy absorption of the material under compression can be calculated from the experimental stress–strain curve. The calculation formula of the energy absorption capacity *C* can be obtained from the following formula:

(4) $$C=\int_{0}^{\varepsilon_{m}}\sigma (\varepsilon )d\varepsilon$$

**Figure 8:**
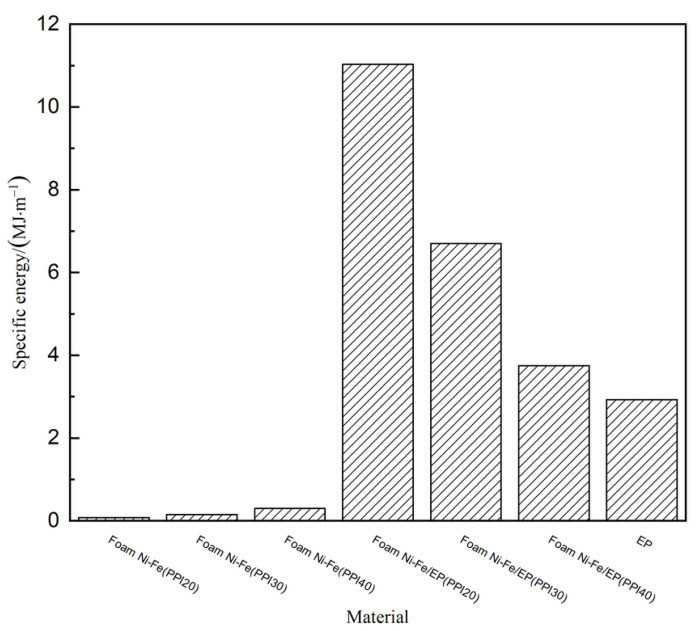
Unit volume energy absorbing of the seven materials.

In Equation (4), *ε_m_* is the maximum strain at the stage of compression to compaction; σ and *ε* represent compressive stress and strain, respectively; *C* is equivalent to the area enclosed by the compressive stress–strain curve when the strain is *ε_m_*. The energy absorption of seven materials can be analyzed by using this formula. Among them, the end point of the platform was used as the calculation node of the energy absorption for three kinds of pure foam Ni-Fe with different PPI and IPC with PPI30 and 40, while the maximum stress point before failure was used as the calculation node for the other two materials without the platform stage. As shown in Figure 8, the endergonic ability of three kinds of foam Ni-Fe/EP IPC were obviously better than pure foam Ni-Fe and epoxy resin; it was even larger than the sum of the energy absorption of pure foam nickel-iron and epoxy resin when they were compressed separately. The reason is that the pure foam Ni-Fe mainly depends on the bending deformation of the pore ribs to absorb energy. For the pore ribs with large slenderness ratio, the bending deformation increased significantly when the energy was small. Epoxy resin was used to fill the pores of the foam Ni-Fe, which formed a lateral constraint on the pore ribs. This greatly improved the energy absorption capacity of the Ni-Fe phase. Furthermore, the nickel-iron phase acts as a hoop for the epoxy phase, which makes the epoxy phase in a three-dimensional compression state; this also improves the energy absorption capacity of the epoxy phase. Combined with the previous mechanical performance analysis, it can be considered that the foam Ni-Fe/EP IPC material is a better dual material with structure and function.

### 3.4. Stress Relaxation Analysis

**Figure 9:**
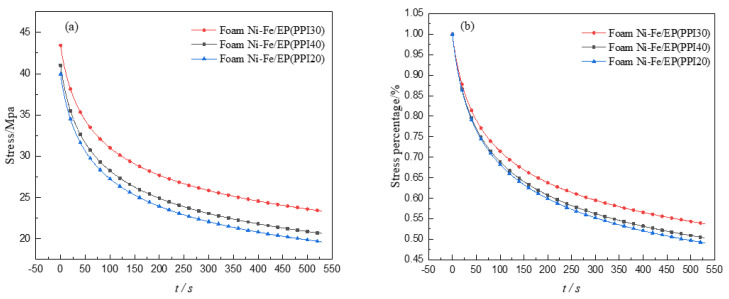
Curves of stress relaxation (**a**) and their percentage of stress relaxation (**b**).

It can be seen from the experiment that the stress relaxation phenomenon appeared in the specimen of foam Ni-Fe/EP IPC material with different PPI. This experiment gives a test force within 530 s after the displacement is kept constant when the amount of compression is 2.5 mm, and the stress relaxation is obtained as shown in Figure 9a. Divided by its initial maximum stress and performing non-dimensional treatment, the relationship curve of the percentage value of the residual stress with time can be obtained as shown in Figure 9b, where the relaxation rate of each specimen can be obtained.

It can be seen from Figure 9a that the three different composite materials all showed an obvious stress relaxation phenomenon, the stress relaxation trend was roughly the same, the stress decreased faster at the beginning, and with the increase in time, the relaxation phenomenon slowed down. The higher the strength of the material, the less stress relaxed in the same time. As can be seen from Figure 9b, the degree of stress relaxation of foam Ni-Fe/EP with PPI20, 30, and 40 was 51%, 46%, and 50%, respectively. It can be seen that with the increase in PPI, the stress relaxation degree of the composite decreased first and then increased in the same time, which indicates that the foam Ni-Fe/EP composite had obvious viscoelastic and plastic unloading capacity.

### 3.5. Creep Analysis

**Figure 10:**
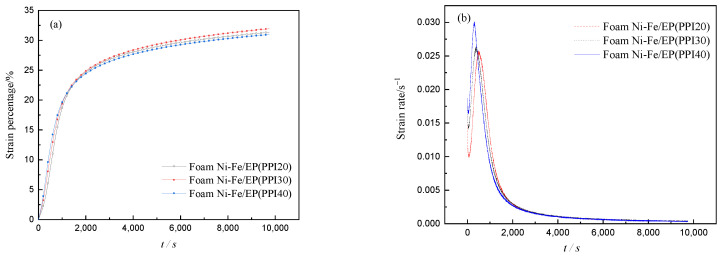
Curves of creep (**a**) and creep rate (**b**).

The creep curves of deformation and time of IPC specimens with different PPI were measured by creep experiments, as shown in Figure 10a. The experimental curves show that the stress remained unchanged when the applied stress was 42 MPa, and the displacement changes around 9800 s were recorded. In addition to the unstable stage at the beginning of creep, it can be seen from Figure 10a that the creep behavior of three different materials mainly manifested in three stages. The first stage is the sharp rise in the creep curve, which represents the elastic deformation of the composite material. The second stage is the transitional creep stage in which the creep curve gradually slows down, representing the viscoelastic deformation of the composites. The third stage is the steady-state creep stage when the creep curve gradually flattens, representing the viscous deformation of composite materials [19]. As can be seen from Figure 10a, the transition creep stage lasts for a long time, indicating that the viscoelasticity of the foam Ni-Fe/EP composite material is obvious. By calculating the slope of each point in Figure 10a and we can get the creep rate, the relationship between the creep rate and time of the three composites in the creep process can be obtained, as shown in Figure 10b. In the whole creep process, except the unstable stage at the beginning of the creep, the creep rate process of the specimen experienced three stages: increase, decrease, and stability. As can be seen from Figure 10b, in the creep elastic stage, the creep rate of the IPC material with PPI40 was the fastest, while that of the IPC material with PPI20 was the lowest. However, in the transition creep stage, the creep rate of the IPC material with PPI 20 was the highest, and that of the IPC material with PPI 40 was the lowest.

## 4. Conclusions

(1) The three-stage deformation characteristics of typical foam materials appeared in the compression process of pure foam Ni-Fe. Foam Ni-Fe/EP interpenetrating phase composites (IPC) have four stages of deformation. The plastic platform stage was very short when PPI was 20, similar to that of pure epoxy resin.
(2) The effective elastic modulus of the three kinds of foam Ni-Fe/EP interpenetrating phase composites (IPC) was smaller than that of the pure epoxy resin, but all were greatly improved than that of the pure foam Ni-Fe. Compared with the pure epoxy resin, the foam Ni-Fe/EP composites with PPI30 and 40 had a plastic platform in the compression process, which was higher than that of the pure foam Ni-Fe. The mechanical properties of the three IPC materials first increased and then decreased with the increase in PPI.
(3) Through the comparison and analysis of the mechanical properties of the three materials such as strength, stiffness, specific strength, and specific stiffness, the foam Ni-Fe/EP interpenetrating phase composites with PPI30 and 40 were better than those of pure foam Ni-Fe and pure epoxy and the energy absorption performance was higher than that of pure epoxy resin and pure foam Ni-Fe. As the PPI increased, the energy absorption rate per unit volume of IPC materials decreased.
(4) The three kinds of foam Ni-Fe/EP IPC materials had obvious stress relaxation phenomenon, and the stress relaxation rate decreased first and then increased with the increase in PPI, indicating that the material had obvious viscoelastic and plastic unloading capacity, which is similar to epoxy resin.
(5) The creep behavior of the three kinds of foam Ni-Fe/EP IPC materials was obvious, and the creep rate increased with the increase in PPI in the creep elastic stage. The creep rate decreased with the increase in PPI in the creep transition stage.

## Figures and Tables

**Table 1:** C_1_ of the foam nickel-iron material.

Materials	Foam Material’s Density ρ* (kg·m^−3^) (10^3^)	Foam Material’s Elastic Modulus E*/Pa (10^6^)	Matrix Material’s Density ρ_s_ (kg·m^−3^) (10^3^)	Matrix Material’s Elastic Modulus E_s_/Pa (10^6^)	Proportionality Constant C_1_
Pure foam Ni-Fe (PPI20)	0.18	3	8.23	89,639	0.06996
Pure foam Ni-Fe (PPI30)	0.21	6	8.23	89,639	0.10276
Pure foam Ni-Fe (PPI40)	0.26	23	8.23	89,639	0.25696
				average	0.14323

**Table 2:** Mechanical properties of the seven kinds of materials.

Materials	Density/(kg·m^−3^) (10^3^)	Elastic Modulus/Pa (10^6^)	Yield Limit/Pa (10^6^)	Specific Stiffness/ (N·m·kg^−1^) (10^3^)	Specific Strength/ (N·m·kg^−1^) (10^3^)
Epoxy(EP)	1.10	390	/	355	/
Pure foam Ni-Fe (PPI20)	0.18	3	0.4	17	2.3
Pure foam Ni-Fe (PPI30)	0.21	6	0.7	28	3.3
Pure foam Ni-Fe (PPI40)	0.26	23	1.4	90	5.5
Foam Ni-Fe/EP (PPI20)	1.43	300	/	210	/
Foam Ni-Fe/EP (PPI30)	1.36	721	55.0	530	40.4
Foam Ni-Fe/EP (PPI40)	1.31	565	36.9	431	28.2

## Data Availability

The data presented in this study are available on reasonable request from the corresponding author.
